# Undesirable Choice Biases with Small Differences in the Spatial Structure of Chance Stimulus Sequences

**DOI:** 10.1371/journal.pone.0136084

**Published:** 2015-08-25

**Authors:** David Herrera, Mario Treviño

**Affiliations:** Instituto de Neurociencias, Universidad de Guadalajara, Guadalajara, Jalisco, México; University of Groningen, NETHERLANDS

## Abstract

In two-alternative discrimination tasks, experimenters usually randomize the location of the rewarded stimulus so that systematic behavior with respect to irrelevant stimuli can only produce chance performance on the learning curves. One way to achieve this is to use random numbers derived from a discrete binomial distribution to create a 'full random training schedule' (FRS). When using FRS, however, sporadic but long laterally-biased training sequences occur by chance and such 'input biases' are thought to promote the generation of laterally-biased choices (*i.e.*, 'output biases'). As an alternative, a 'Gellerman-like training schedule' (GLS) can be used. It removes most input biases by prohibiting the reward from appearing on the same location for more than three consecutive trials. The sequence of past rewards obtained from choosing a particular discriminative stimulus influences the probability of choosing that same stimulus on subsequent trials. Assuming that the long-term average ratio of choices matches the long-term average ratio of reinforcers, we hypothesized that a reduced amount of input biases in GLS compared to FRS should lead to a reduced production of output biases. We compared the choice patterns produced by a 'Rational Decision Maker' (RDM) in response to computer-generated FRS and GLS training sequences. To create a virtual RDM, we implemented an algorithm that generated choices based on past rewards. Our simulations revealed that, although the GLS presented fewer input biases than the FRS, the virtual RDM produced more output biases with GLS than with FRS under a variety of test conditions. Our results reveal that the statistical and temporal properties of training sequences interacted with the RDM to influence the production of output biases. Thus, discrete changes in the training paradigms did not translate linearly into modifications in the pattern of choices generated by a RDM. Virtual RDMs could be further employed to guide the selection of proper training schedules for perceptual decision-making studies.

## Introduction

In typical two-alternative discrimination tasks, subjects are required to make choices between two options offered [1–4]. They receive rewards after correct responses on those trials in which they respond to the side where the 'positive' discriminative stimulus (S^D^) appears, whereas they are credited with errors when they choose the incorrect stimulus on the other side (*i*.*e*. the stimulus delta, S^Δ^). A major problem for the data analysis from these kind of experiments involves deciding whether fluctuations in the discrimination learning curve away from chance represent genuine changes in the subject's behavior with respect to the S^D^, or whether they derive from some behavior that responds some other features of the situation (*v*.*gr*. the reward history) [4–8]. Thus, aiming to improve their measurements, experimenters commonly randomize the locations occupied by the rewarded stimuli, so that systematic behavior with respect to irrelevant stimuli/factors can only produce chance performance on the learning curve [9]. By definition, these presentation series contain an equal number of opposing sequences, balancing the frequency of reward for both options.

A practical way to create a 'full random training schedule' (FRS) for discrimination tasks is to use random numbers derived from a discrete binomial distribution. When using FRS, however, long but sporadic training sequences, in which reward is placed in the same location, occur by chance. We will refer to such repeating chunks of laterally-biased training sequences as 'input bias'. To justify such an unconstrained training sequence generation, one must either assume that the subjects are not influenced by input biases or that the effects of input biases are washed out over time. Yet, empirical evidence indicates that during the early training phase subjects do not immediately link their responses to the S^D^ and they also tend to establish behavioral strategies based on recent reward history [2–4,10]. For these reasons, choosing the right order and combination of discriminative stimuli during training is extremely important, particularly when the stimulus trials are larger than the number of display locations (two for dichotomic tasks). For example, the number of trials in which the same comparison stimulus is scheduled as correct can induce temporary stimulus preferences that interfere with the reinforcement contingencies intended by the experimenter, whereas disruptive location preferences can result from repeated presentations of correct stimuli at the same location [10–13].

One effort to restrain the source of such input biases for training sequences is the 'Gellerman-like training schedule' (GLS; [2–4,14]), a template designed to render the S^D^ on each trial unpredictable from the outcome of previous trials, such that its location is not correct for more than three consecutive times. In this way, obvious artifacts such as alternating sequences are not introduced.

Here, we compared the choice patterns produced by a virtual 'Rational Decision Maker' (RDM) in response to computer-generated FRS and GLS training sequences. Through computer simulations, we found that, although the GLS presented fewer input biases than the FRS, the virtual RDM produced more steady-state output biases with GLS than with FRS under a variety of test conditions. This confirms the fact that the statistical and temporal properties of training sequences interacted with the RDM to influence the production of output biases.

## Results

Although it is clear that FRS contains an implicit source of input biases, we do not understand exactly how switching from FRS to GLS affects the production of output biases produced by a simple 'Rational Decision Maker' (RDM; see equations below). This is a very relevant question because choice biases are deleterious for experiments in psychophysics: they affect the estimation of perceptual thresholds [3,4,6], are associated with higher error rates, less reinforcement and also usually reflect a general disengagement from animals performing the behavioral task [1,4,6,15].

Reinforcement learning theories provide a powerful theoretical framework for the description of choice behavior in dynamic environments: they state that future actions are chosen to maximize a long-term sum of positive outcomes, which can be accomplished through a set of value functions that represent the amount of expected reward associated with particular states or actions [13]. To match behavior to income, animals must integrate the rewards earned earlier from particular behaviors, and maintain an appropriate representation of the value of competing alternatives (*i*.*e*. reward frequency). Quantitatively, this idea is captured by the matching law [16,17], which states that the long-term average ratio of choices matches the long-term average ratio of reinforcers [16,18,19]:

$$\frac{C_{R}}{C_{L}}=c{\left(\frac{R_{R}}{R_{L}}\right)}^{a}$$ (1)

where *C*_*R*_ and *C*_*L*_ denote the number of steady-state responses, and *R*_*R*_ and *R*_*L*_ the number of steady-state reinforcers for the right (*R*) and left (*L*) options, respectively. The coefficient *a* denotes the sensitivity to the reinforcement ratio, and *c* is a bias term unrelated to reinforcer frequency or magnitude [18,19]. This equation describes an empirical regularity of behavior in which the average tendency of subjects to make their choices is in correspondence with the frequency of reward they receive from choosing it. It predicts that the amount of laterally biased choices (output biases) should be proportional to the amount of laterally biased rewards (input biases).

The reward history from a S^D^ influences the probability of choosing it again on subsequent trials [4,10–13,20]. Because location habits are a common feature of animal behavior [4,21], it is crucial to carefully choose the training sequences of locations for the S^D^. Such sequences should prevent better, or worse, than chance performance arising from systematic behavior based upon location, and should also prevent the accidental reinforcement of any such behavior.

Typically, FRS have been used to reduce the predictability of reward, aiming to make discrimination tasks independent of spatial cues. However, FRS contain a source of input biases which may influence the production of output biases. Thus, it would be desirable to test GLS instead of FRS because the former has fewer input biases.

By assuming that choices comply with matching behavior, we hypothesized that the reduced number of input biases in GLS would decrease the production of output biases compared to FRS. Such a reduction could increase the task efficiency because biased choices are linked to errors (in balanced tasks). If true, this would suggest a positive interaction between the GLS and the decision-making process. If not, it could either reflect a non-linearity in the decision-making process or that biased choices do not comply with matching behavior.

**Fig 1:**
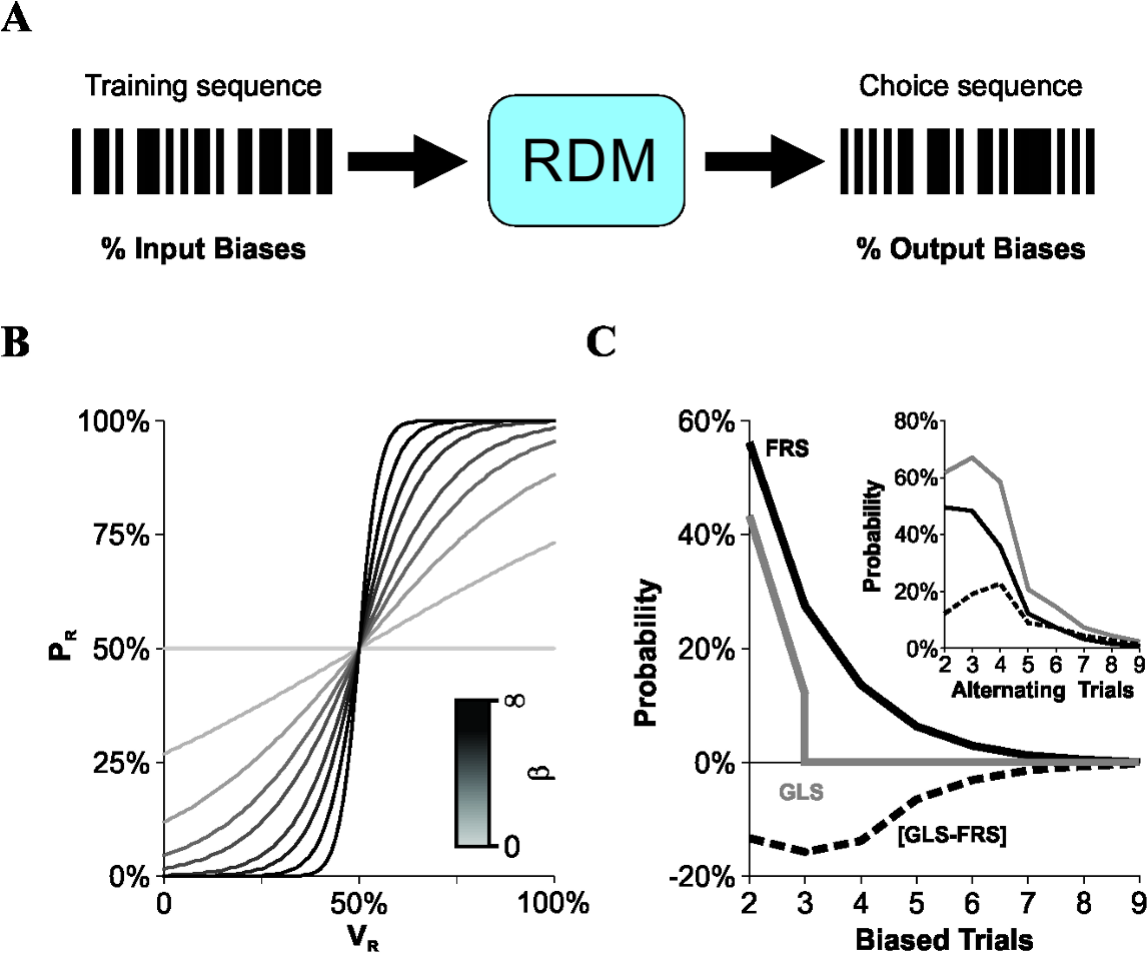
Virtual decision maker and training schedules with different input bias probabilities. (A) A training sequence with a % input biases is processed by a virtual 'Rational Decision Maker' (RDM) to produce choice sequences with a % output biases. (B) The RDM algorithm varied according to a sigmoidal function, in which the probability of choosing to the right (*P*_*R*_, y-axis) depended on the weight function for the right option (*V*_*R*_, x-axis) and on the slope of the curve (*β*; darker lines represent higher *β* values; Eq 2). (C) The probability of finding laterally biased sequences (y-axis) of different lengths (x-axis) is smaller using GLS (gray line) than FRS (black line). The opposite occurred for alternating sequences (inset). The differences between group probabilities are displayed by dotted lines and are always negative. We calculated the probabilities for biased and alternating sequences by creating independent sets of randomized sequences. Average probabilities for biased/alternating 2-trial-sequences with the FRS schedule converge towards 50% when the number of trials is increased (*v*.*gr*. from 100 to 1000; not illustrated).

Our specific aim was to compare the choice patterns produced by a RDM in response to FRS and GLS training schedules (Fig 1A). First, we made linear arrays of pseudo-random numbers derived from a discrete binomial distribution to create FRS and GLS training schedules (100 trials x 100 repetitions). The GLS algorithm was identical to the FRS one, but it additionally prohibited sequence repetitions of more than three consecutive trials [2–4,14]. Reinforcement was provided on each trial for one of the two mutually exclusive options (*i*.*e*. probability of reward per trial = 100%; probability of reward per side = 50%). To create a virtual RDM, we implemented an algorithm that made choices based on past rewards and choices according to an exponentially-weighted moving average filter (EWMA), but was insensitive to differences in discriminative input signals [1]. With this model, we assumed that the integration of past rewards is imperfect (*i*.*e*. leaky), which translates into a finite effective memory on estimates of income, making them local rather than global (in time) [13]. Indeed, the EWMA provides a description for short-term memory in which a reinforcer produces smaller effects into current choices as one considers responses that extend further into the past [12,22]. We chose to use the EWMA based on a series of quantitative observations made on the choice records from diverse animal models [4,11,13,19,23], and because it has fewer free parameters than other alternative models *v*.*gr*. [24]. 'Memory gradients' have been also described with hyperbolic functions [19,25], but this won't be addressed here.

The probability that the RDM chose a particular option was computed by comparing a uniformly distributed pseudo-random number against a logistic curve with the following form:

$$P_{R}=\frac{1}{1+e^{\beta \left(V_{R}-V_{L}+B\right)}}$$ (2)

where *β* corresponds to the slope of the sigmoid (Fig 1B, darker lines represent higher *β* values; see also [26,27]), and *V*_*R*_ and *V*_*L*_ are weight functions for the right and left options, respectively, and *B* is a positive or negative constant (with randomized sign) that can be used to represent individual biases towards one or other option (*i*.*e*. a lateral bias, hereafter referred to as 'intrinsic laterality' [28]). In this system, *V*_*R*_ and *V*_*L*_ served to horizontally shift *P*_*R*_ towards one or the other option, influencing future choices. Also, the weight functions for the chosen (*x*) and un-chosen (*y*) options were updated on a trial-by-trial basis using the Euler approximation of derivatives:

$$v_{x,i}=\{\begin{array}{l} 1\;\text{if rewarded}\;(\text{a})\\ k_{1}∙v_{x,i-1}\;\text{if not rewarded}\;(\text{b}) \end{array}$$ (3)

v_(y,i)=k_2〖∙v〗_(y,i−1)if un-chosen (4)

According to these equations, every time a choice was rewarded, its corresponding weight value was increased to one (Eq 3(A)), whereas the weight values for the un-rewarded (Eq 3(B)) and the un-chosen (Eq 4) options decreased (1−*k*_1_) and (1−*k*_2_), respectively (on every trial). These *k* values are directly linked to a mono-exponentially decaying process with a *τ* = −1/ln(*k*). An initial fast reduction of the weight value of the un-rewarded option (small *k*_1_), followed by a slower reduction in the weight value for the un-chosen option (*k*_2_ > *k*_1_) are consistent with the law of effect [28,29] and could be a plausible representation of short-term memory [11].

To quantify the amount of biased and alternating sequences in the training and choice records, we implemented a pair-wise alignment method that consisted in sliding each query sequence along the record of interest. A sequence was considered to be present in the record when the alignment matched perfectly for the entire length of the query sequence (*i*.*e*. sequence similarity = 1). The average probability of occurrence of a sequence was then calculated by dividing the number of times that it was found in the record by the maximum number of times that it could fit within the total length of the record without interfering with any identical sequence that generated a count in previous trials. Note that comparing the average probabilities instead of the number of cases is fundamental because the alignment method implicitly increases the counts for shorter sequences contained in longer ones [4]. With this tool, we next analyzed the input bias probability for complementary sequences using training trials produced by both sequence-generation algorithms and found that, as expected, it was smaller for GLS (gray line) than for FRS (black line; Fig 1C). Notably, biased sequences with a length of two and three trials were also more likely to occur in FRS than in GLS, making the overall difference in input bias probabilities always negative (Δ*P = P*_*input_bias[GLS]*_ *—P*_*input_bias[FRS]*_; dotted line; Fig 1C). Furthermore, because the probability of finding biased sequences of length > 3 in GLS is always zero, this implies that the difference between these distributions must always be negative. Such a difference could mean that other un-biased sequences must have changed their probability of occurrence. We counted the number of complementary alternating sequences from the same training schedules and found that they were indeed more frequent in GLS (gray line) than in FRS (black line; Fig 1C, inset). Thus, switching from FRS to GLS fully re-arranged the probabilities of observing biased and alternating sequences during training. However, the reward probability in steady state was well balanced between the two options for both groups (FRS: 50.66% ± 0.50% *n* = 10,000; GLS: 50.00% ± 0.50% *n* = 10,000). Note that we calculated the probabilities for biased and alternating sequences by creating independent sets of randomized sequences (100 repetitions).

**Fig 2:**
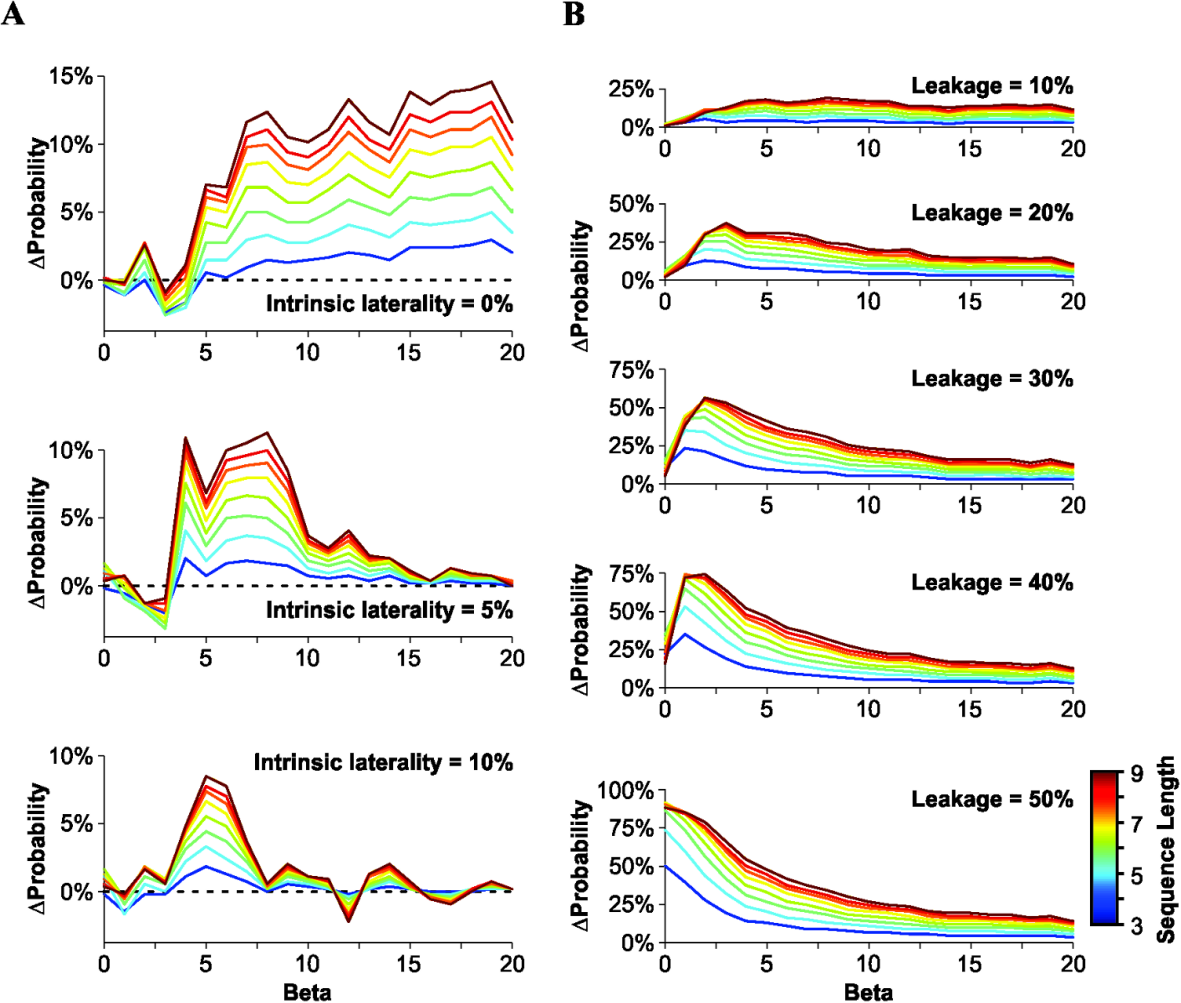
Training the RDM with FRS and GLS. Results are displayed as the difference in the probability of finding output biased choices produced with a 'Gellerman-like' (GLS) minus a 'Full Random' (FRS) training schedule, after being processed by the 'Rational Decision Maker' (RDM) algorithm (*i*.*e*. *ΔP = P*_*output_bias[GLS]*_ − *P*_*output_bias[FRS]*_). *ΔP* values were computed as a function of *β* (x-axis; Eq 2) and of sequence length (from 3 to 9; darker lines represent longer sequences). *ΔP* values were always positive (for *β* ≥ 3), yet they decreased with intrinsic laterality (A; *k*_*1*_ = 0.5, *k*_*2*_ = 0.5, leakage = 0%), but increased with leakage (B; *k*_*1*_ = 0.5, *k*_*2*_ = 0.5, intrinsic laterality = 0%).

We next asked how GLS and FRS influenced the production of output biases when processed by the same virtual RDM. To avoid misestimating output probabilities, we tested the RDM with 100 trial sequences that were re-randomized according to GLS and FRS for every condition. We mapped for a variety of internal mechanisms of the RDM: multiple *β* values, intrinsic laterality and different *k* values. For this analysis, we measured the probability of occurrence of output biases of variable length dividing the number of times that each biased sequence was found in the choice record by the maximum number of times that it could fit in the choice record if the choices had been completely biased. To facilitate the visualization of results, we subtracted the output bias probability obtained when using GLS from that one produced when using FRS (*ΔP = P*_*output_bias[GLS]*_ − *P*_*output_bias[FRS]*_). Surprisingly, the output bias probability was higher with GLS than with FRS (for *β ≥* 3), and group differences increased with the length of the biased sequence (darker lines for output biased sequences of longer length; Fig 2A upper panel). We approximated the slopes of the fitted logistic curves (*i*.*e*. *β*) taken from normalized data from published psychometric responses from the auditory system in rats and humans (*β* ≈ 12.6, *β* ≈ 13.9, respectively; [29]), and from the visual (*β* ≈ 8.9; [30]) and somatosensory (*β* ≈ 8.4; [31]) systems in monkeys. All these *β* values were bigger than 3. These numbers indicate that our results might have a relevant impact for the estimation of psychometric curves.

To investigate whether the sign of *ΔP* depended on the intrinsic components of the RDM, we introduced a source of individual laterality with fixed magnitude but variable polarity (Eq 2). This number represents an implicit preference of the RDM for a particular option, at the individual but not at the population level (see also [4]). Notably, although *ΔP* decreased with higher intrinsic laterality values, the production of output biases was still larger when using GLS compared to FRS (Fig 2A; middle panel, intrinsic laterality: 5% and lower panel, intrinsic laterality: 10%).

Next, we asked whether an imperfection in the decision-making process could reverse the differences observed in the production of output biases. We introduced a graded leakage in the effective probability of choosing a particular option (*EP*_*R*_ = *[ε∙P*_*R*_ *];* [32]) but found that *ΔP* remained positive for all values tested, and even displayed an increased output bias production with bigger information leakage (Fig 2B).

**Fig 3:**
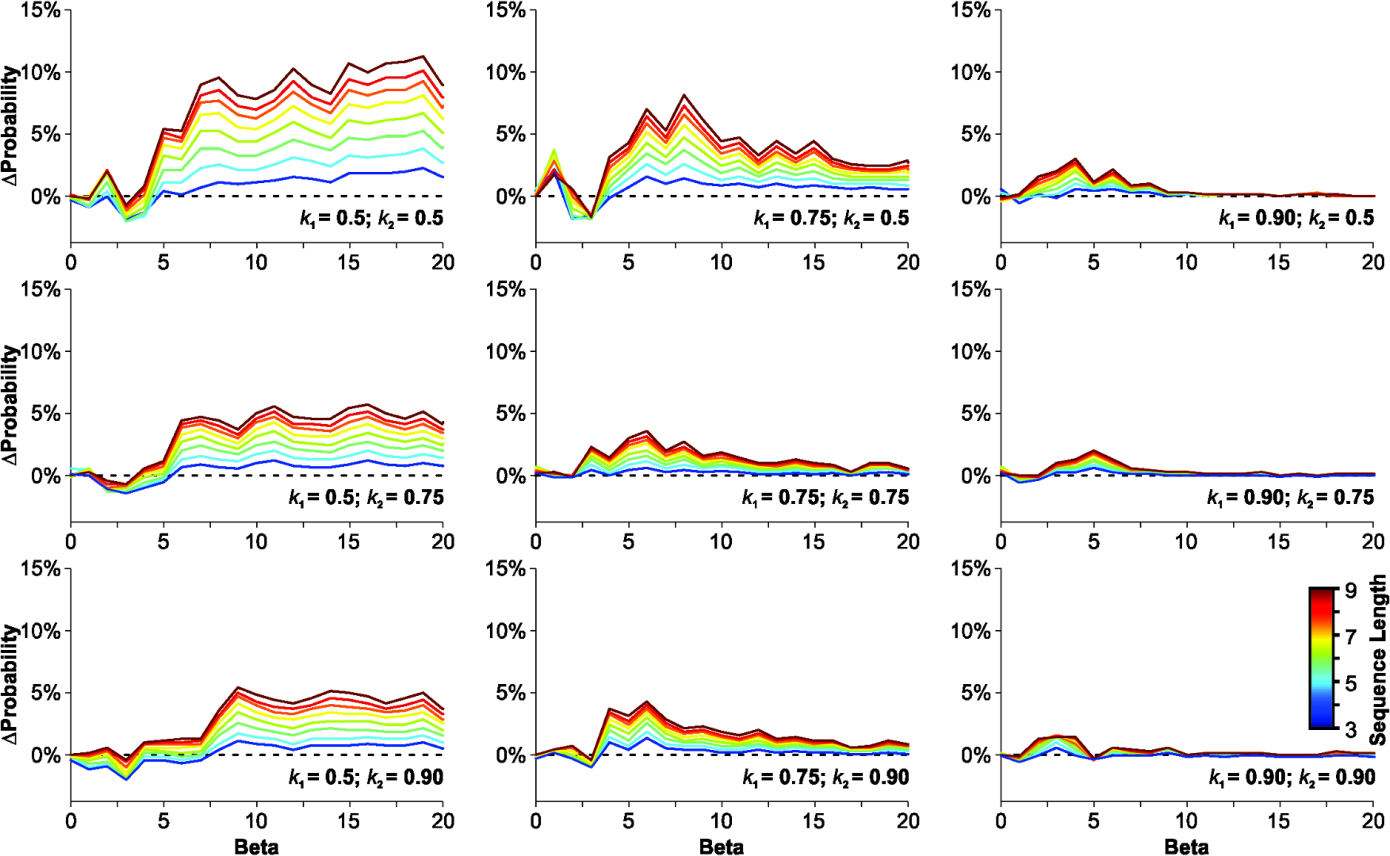
Testing the RDM with different 'short-term memory' decaying rates. Results are displayed as the difference in the probability of finding output biased choices produced with a 'Gellerman-like' (GLS) minus a 'Full Random' (FRS) training schedule, after being processed by the 'Rational Decision Maker' (RDM) algorithm (*i*.*e*. *ΔP = P*_*output_bias[GLS]*_ − *P*_*output_bias[FRS]*_). Panels are arranged in columns (*k*_1_ = 0.5, 0.75 and 0.90) and rows (*k*_2_ = 0.5, 0.75 and 0.90). Increasing *k* values implies longer short-term memory representatioτns. *k* values of 0.5, 0.75 and 0.90 correspond to a *τ* of 1.44, 3.47 and 9.49, respectively.

According to the matching law, each reward contributes equally to increasing the probability of choosing an option [16]. This contribution is conceptualized as being stable over time because these mathematical descriptions are valid only for choice behavior in a steady-state scenario [12]. In a dynamic system, however, the reward changes its location over time and choice behavior is strongly influenced by recently obtained reinforcers [12]. In such scenario, *k*_*1*_ and *k*_*2*_ represent constants that determine the decay rate for the contribution of past rewards to current choice (Eq 3(B) and Eq 4; see also [13]). Higher *k* values imply a higher impact of reward history on current choice, whereas lower values represent a more prolonged effect of reinforcement history. Based on this idea, we investigated whether *ΔP* could invert its sign after changing the relative contribution of past rewards. We tested all possible combinations for three different discrete values for *k*_*1*_ and *k*_*2*_, respectively. In Fig 3 we show that *ΔP* values were positive for all combinations tested (covering both *k*_*1*_> *k*_*2*_ and *k*_*1*_< *k*_*2*_), yet group differences became smaller as *k* values increased. Also, *k*_*1*_ had a stronger effect (than *k*_*2*_) in dissipating *ΔP* for high *β* values (Fig 3).

**Fig 4:**
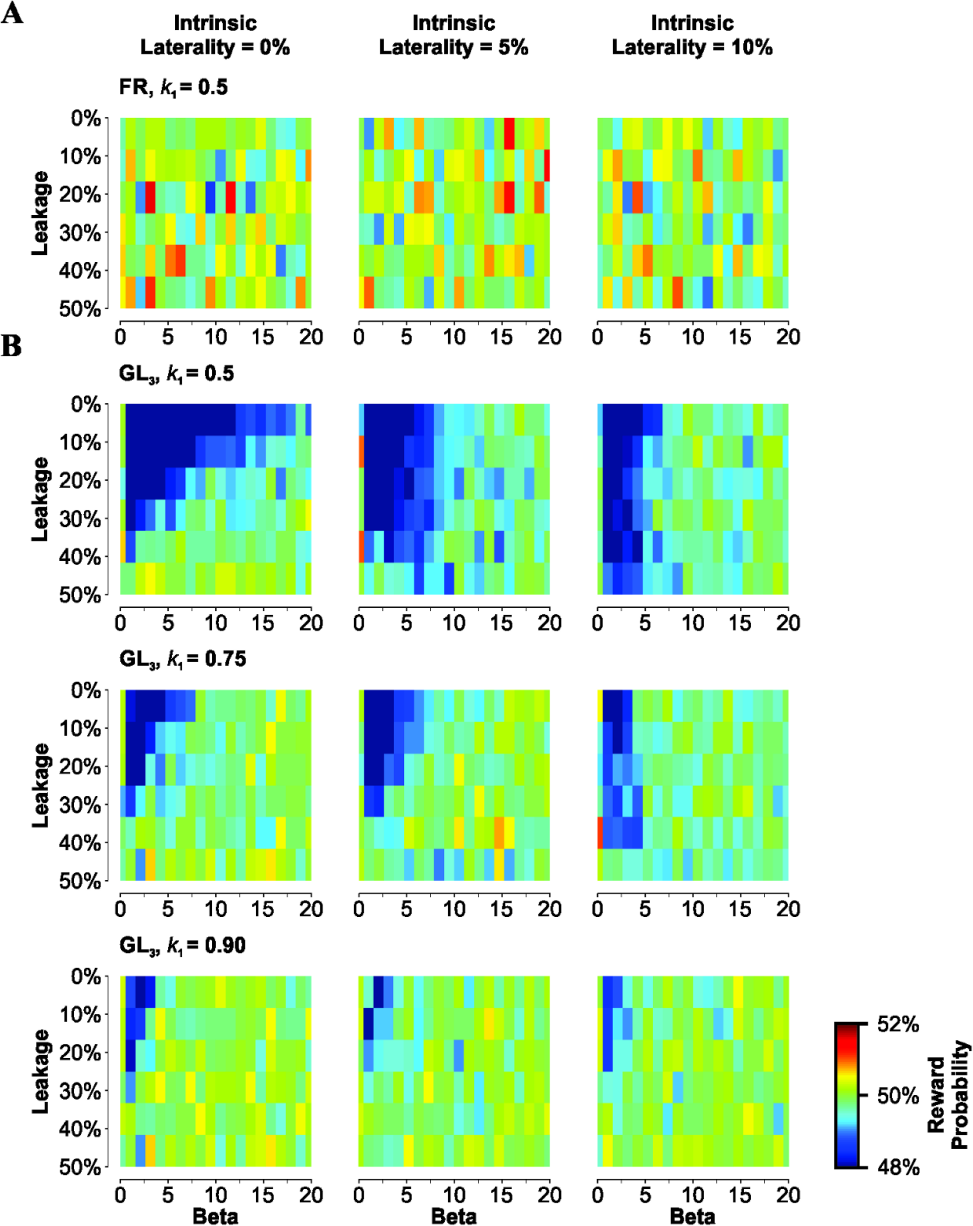
Reduced reward probability when training the RDM with GLS. Reward probability was estimated by computing the group average correlation between reward location and the side to which the choices were made. (A) Irrespective of leakage (y-axis) and *β* (x-axis) values, the reward obtained with a 'Full Random' training schedule (FRS) processed by the 'Rational Decision Maker' (RDM) with increasing intrinsic laterality values (displayed in columns) was always around chance level (50%). (B) There is an apparent drop in reward (dark regions) obtained with a 'Gellerman-like' training schedule (GLS) when processed by the RDM for small leakage and *β* values. However, the drop tends to disappear upon increasing information leakage (y-axis), intrinsic laterality (columns) and increasing *k*_*1*_ (rows; *k*_2_ = 0.5 for all panels).

Because output biases were generally higher with GLS than with FRS, we pondered whether these group differences also led to differential rewards obtained by the RDM. Reward was calculated by using the correlation between reward location and the corresponding choice record from the RDM. As expected for a balanced reward distribution, the reward probability was near chance level when the RDM was trained with the FRS (Fig 4A). When using GLS, however, the reward probability dropped systematically from this level by approximately 2–3% for *β* ≤ 15 and with an information leakage ≤ 30 (dark regions in panels from Fig 4B). This apparent reduction in reward probability vanished upon increasing *β* (x-axis), the information leakage (y-axis), the intrinsic laterality (columns in Fig 4), and also upon increasing *k*_1_ (rows in Fig 4).

**Fig 5:**
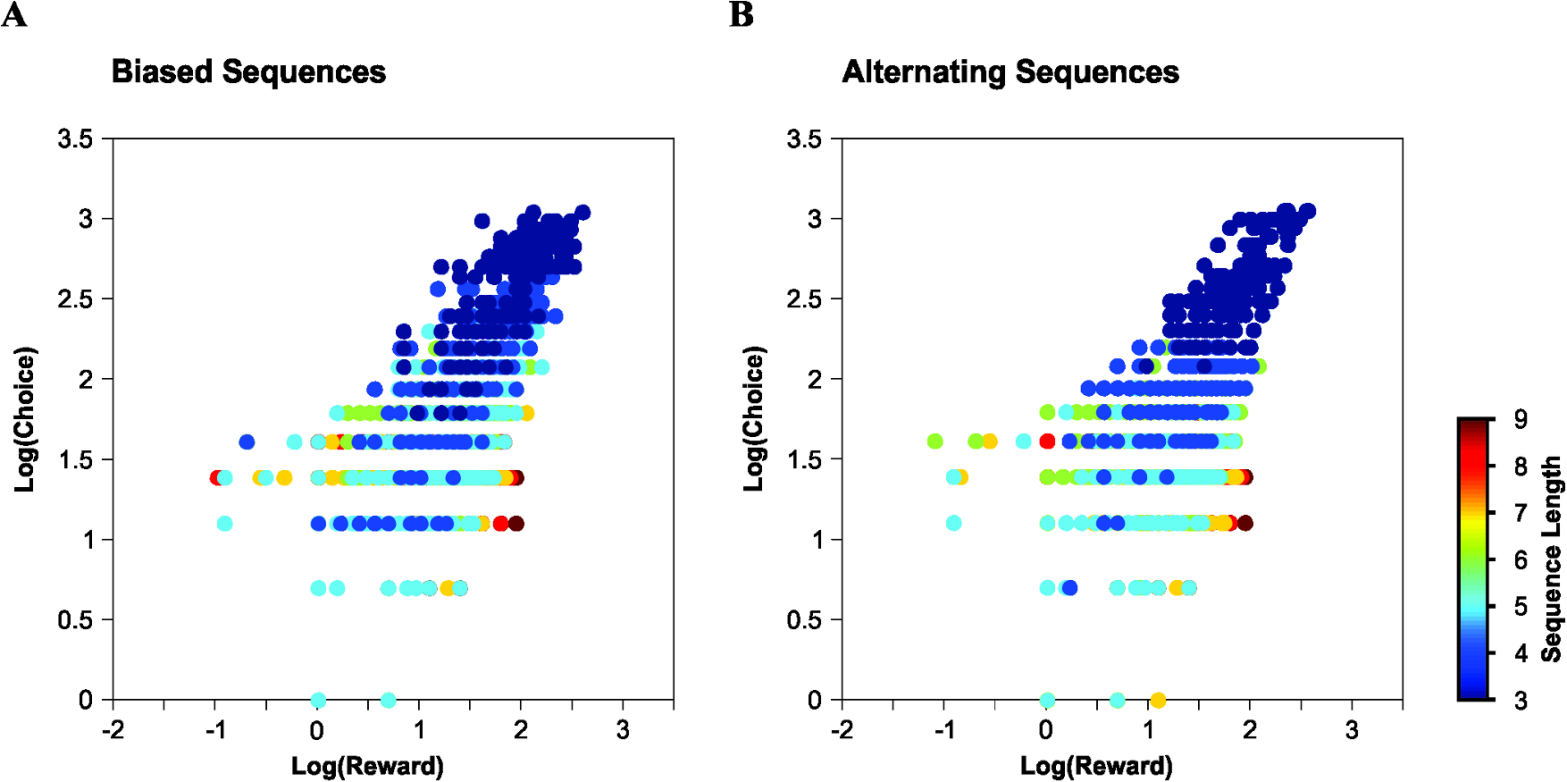
Output choice biases comply with the generalized matching law. The log frequency of choices is linearly related to the log frequency of rewards for biased sequences (A) and alternating sequences (B) obtained with both a 'Full Random' (FRS) and a 'Gellerman-like' (GLS) training schedules processed by the 'Rational Decision Maker' (RDM). Each point represents the frequency of occurrence of a particular choice sequence *versus* the sum of reward values obtained from choosing those specific sequences.

Finally, we probed how the drop in reward probability obtained with GLS training related to changes in the output bias probability. In this case, we selected blocks of 30 training trials and counted the number of times that each sequence occurred within the choice record for each training repetition of the RDM (*i*.*e*. sequence frequency), and calculated the corresponding reward value as the sum of the correct choices for those found sequences (*i*.*e*. reward frequency). Fig 5 shows the log frequency (y-axis) for biased (Fig 5A) and alternating (Fig 5B) choice sequences as a function of the log net amount of reinforcer received (x-axis). The slopes for both models were positive indicating that all biased choices produced by the RDM complied with the matching law.

## Discussion

In two-alternative discrimination tasks, researchers usually present the positive stimulus (S^D^) on either the right or left side of the apparatus according to a fully random training schedule (FRS). It is assumed that such orders of training stimuli provide the subjects an opportunity of making only half of their responses correct through 'chance' alone. In actual experience, however, it has been found that additional factors such as habits of alternation may result in an accuracy higher than 50%. The uncertainty in the interpretation of such results is in part due to the selection of the orders of training stimuli. Gellerman (1933) argued that the FRS allows various incorrect habits, such as location alternation, to produce an accuracy of performance as high as 70%. He attributed the general uncertainty on where to put the criterion for learning to the faulty selection of orders of alternating stimuli [14]. To remedy this situation, he proposed some criteria necessary to ensure balanced random sequences, as in the 'Gellerman-like training schedule' (GLS) that we used here. Indeed, the GLS removes input biases by prohibiting the S^D^ from appearing at the same location for more than three consecutive trials [2–4,21].

Although it was clear to us that the FRS contained an implicit source of input biases, we could not predict how switching from FRS to GLS training scheme would affect the production of output biases. We thus created a virtual 'Rational Decision Maker' (RDM) that was sensitive to past rewards, trained it with FRS and GLS sequences, and measured its response properties under a variety of test conditions by changing *β*, intrinsic laterality, information leakage and *k* values. Unexpectedly, although GLS contained fewer input biases than FRS, the RDM produced more output biases when trained with GLS rather than with FRS (for *β* ≥ 3). Interestingly, we introduced an internal source of intrinsic laterality [4] and found that it contributed in dissipating the differences in output biases between groups. One possibility is that such differences were still existent but became much smaller, because intrinsic laterality increased the portion of biased choices that are independent of the training schedule. Moreover, since alternating choices increase with smaller *β* values, this should reduce the relative impact of the intrinsic laterality in the choices made by the RDM. Also, regardless of the relative differences in the production of output biases between FRS and GLS (*i*.*e*. a between-group comparison), we confirmed that all biased sequences produced by the RDM complied with the matching law (*i*.*e*. a within-subject relationship).

The output choices of the RDM depended on a dynamic competition between weight values for both options (*V*_*R*_ and *V*_*L*_) that were raised when rewarded but decayed for un-rewarded and un-chosen options. When the weight values were similar (*i*.*e*. *V*_*R*_ */V*_*L*_ ≈1), the decision process tended to be at random, favoring alternation between options. In contrast, when the weight value for one option grew relative to the opposite one (*v*.*gr*. *V*_*R*_>>*V*_*L*_; *V*_*R*_ */V*_*L*_ → ∞ and *V*_*R*_ */V*_*L*_ → 0), the likelihood of choosing the former option increased (*P*_*R*_ *>>P*_*L*_), creating a tendency in the RDM to produce more output biases. Accordingly, to promote alternation in the RDM, it was necessary to decrease the weight value of the favored option (by not rewarding it) while increasing the weight value of the opposite option (by rewarding it) until a *V*_*R*_ */V*_*L*_ ≈1 balance was reached.

Weight values for un-rewarded and un-chosen options decayed mono-exponentially at different rates, which we mapped for different values. However, irrespective of which of these functions decayed faster, the RDM always produced more output biases with GLS than with FRS. Indeed, for both weight value representations, smaller *k* values led to a faster decay rate that promoted alternation, whereas a bigger values lead to a longer integration time window, making the choices of the RDM (*i*.*e*. output) less sensitive to the local (temporal) variations in training conditions (*i*.*e*. input). Thus, the decay time constants of the un-rewarded and un-chosen options strongly determined the period during which the RDM was sensitive to the input stream of training sequences. Clearly, the fastest way to remove an output bias in the RDM would have been by using an input bias towards the opposite option, as has been suggested previously [2–4]. The lack of input biases in GLS implied that long sequences of biased choices could not be rewarded (and un-rewarded; *P*_*R*_ = *1-P*_*L*_), thus reducing the efficiency of GLS, compared to FRS, to counterbalance the emergence of output biases. In contrast, the naturally occurring input biases in FRS provided the right opportunity to produce sequences of un-rewarded choices, which contributed to adjust the weight values, reduce output biases and promote alternation. Thus, the GLS training sequence contains information about which stimulus will be the rewarded one, at least in cases where three repetitions have occurred already. Because the RDM is sensitive to past rewards, it might seem then less surprising that the GLS led to more output biases than the FRS.

One important limitation of our study is that all the results presented here are strictly dependent on: i) the equations that we used to describe the RDM and ii) the fact that these equations successfully describe, by using very few free parameters, some basic principles of animal choice behavior [12,13,19,24]. However, depending on how the RDM diverges from real animal choice behavior, the results could lose their potential relevance. It is thus crucial to test these predictions with animal models. We have started doing some attempts in this direction [4]. Using GLS for training, we have shown that, depending on the degree of discriminative information, mice display different amounts of biased choices [1]. In conditions of high discriminability, the mice learned to discriminate and produce a reduced number of side-biased choices. However, as discriminability decreased, they increased the usage of biased choices to solve the task [4]. Thus, when the discriminative information was low, the reward history became the most important cue for guiding their behavior [4,10,11]. An interesting possibility is that such biased choices could constitute a valid strategy for solving tasks when discriminative cues are scarce. These observations suggest that output biases could indeed be modulated by quantitative differences in the discriminability of sensory stimuli. How exactly the discriminative information regulates the strength of the dependency on past rewards (and choices [12]) to produce biased choices remains an open question.

The GLS has been a standard for decades in research on discrimination learning [14]. Using numerical simulations, we here showed that a simple virtual RDM unexpectedly produced more (and not fewer) biased choices when trained with GLS instead of FRS. Thus, it might be desirable to train with GLS but switch to FRS once the subjects reach a steady-state choice behavior. Certainly, as the complexities of the training procedures increase, the challenges in making appropriate combinatorial decisions concerning stimulus order and location sequences become progressively greater [9,33].

Our results demonstrate that the statistical and temporal properties of training streams interact with the internal components of the RDM to influence the production of output biases. They highlight the fact that discrete changes in the training paradigms do not translate linearly into changes in the output choices made by this simple choice maker. Therefore, manipulations of the properties of input streams must be made with extreme precaution because they may have a relevant impact on the estimation of psychophysical measures that are assessed through choice behavior. We propose the usage of virtual RDMs to select appropriate training streams for perceptual decision-making studies.
