# Correction: Undesirable Choice Biases with Small Differences in the Spatial Structure of Chance Stimulus Sequences

**DOI:** 10.1371/journal.pone.0139259

**Published:** 2015-09-22

**Authors:** The PLOS ONE Staff

In the Results section, there is an error in the fourth equation. The publisher apologizes for this error. The correct equation is:

$$v_{y,i}=k_{2}\cdot v_{y,i-1}\text{ if un-chosen}$$ (4)
